# One-leg standing time is a simple measure for loss of skeletal muscle mass and fat deposition in muscle: the J-SHIPP study

**DOI:** 10.1007/s40520-023-02665-8

**Published:** 2024-01-28

**Authors:** Yasuharu Tabara, Yoko Okada, Masayuki Ochi, Yasumasa Ohyagi, Michiya Igase

**Affiliations:** 1 Graduate School of Public Health, Shizuoka Graduate University of Public Health, Kita-Ando 4-27-2, Aoi-Ku, Shizuoka, 420-0881 Japan; 2 Center for Genomic Medicine, Kyoto University Graduate School of Medicine, Sakyo-Ku, Kyoto, 606-8507 Japan; 3 Department of Geriatric Medicine and Neurology, Ehime University Graduate School of Medicine, Toon City, 791-0295 Japan; 4 Department of Anti-Aging Medicine, Ehime University Graduate School of Medicine, Toon City, 791-0295 Japan

**Keywords:** One-leg standing time, Muscle mass, Muscle quality, Older adults

## Abstract

**Backgrounds:**

One-leg standing time (OLST) has been frequently used physical performance measure; however, what muscular characteristics OLST represents remains uncertain.

**Aim:**

This cross-sectional study aimed to investigate the association between OLST and muscle characteristics to clarify the possibility of using OLST as a physical performance measure.

**Methods:**

Study participants comprised 1144 older adults aged 65 years or older. Computed tomography images provided mid-thigh skeletal muscle cross-sectional area and mean attenuation value. OLST was measured for a maximum of 60 s. Static postural instability was assessed using a posturography.

**Results:**

A frequency of OLST < 20 s was increased by quartiles of muscle cross-sectional area (Q1: 33.6, Q2: 12.8, Q3: 13.6, Q4: 11.9%, *P* < 0.001) and mean attenuation value (Q1: 32.3, Q2: 21.7, Q3: 14.3, Q4: 7.7%, *P* < 0.001). Results of the multinomial regression analysis indicated that muscle cross-sectional area and mean attenuation value were independently associated with an OLST of less than 20 s. The crude odds ratio of OLST less than 20 s for the lowest quartiles of both cross-sectional area and mean attenuation value was 4.19 (95% CI: 3.01 − 5.84). The cross-sectional area of muscles with greater fat deposition was inversely associated with OLST, while that with smaller fat deposition showed a positive association with OLST, indicating why mean attenuation value and cross-sectional area were independently associated with OLST. No clear relationship was observed with static postural instability.

**Conclusion:**

OLST was a simply measurable quantifiable physical measure representing the loss of muscle mass and quality in older adults.

**Supplementary Information:**

The online version contains supplementary material available at 10.1007/s40520-023-02665-8.

## Introduction

In addition to low muscle mass and weak muscle strength, low physical performance measures were independently associated with a future decline in daily-living activity [1]. Among several measures of physical performance, gait speed has been usually used to assess sarcopenia [2, 3]. However, because gait speed measurement requires around 10 m of walkways, it is not easy to measure it in clinical setting or at the bedside. Given this issue, the revised guidelines from Asian Working Group of Sarcopenia adopted the chair stand test as a physical performance measure [4]. The chair stand test has also been adopted in the European guidelines as one of the tests of short physical performance battery [3, 5]. One-leg standing time (OLST) was another simple measure for physical performance that can be assessed at bedside without specific device. Several studies reported shorter continuous time of OLST as a risk factor for hip fractures [6, 7], a decline in activity of daily living [1, 7], and all-cause mortality [8]. These findings support the possibility of using OLST as another physical performance measure, although in order to adopt OLST, the factors determining OLST need to be clarified. Several factors, such as asymptomatic brain damage [9], cognitive decline [9], and impairment of sensory, motor, and central processing systems [10], influence OLST. Furthermore, low appendicular muscle mass [11, 12] may also be a factor for decreasing OLST; however, no studies have investigated the possible involvement of directly measured muscle quality (i.e., amount of fat deposition in the skeletal muscle) in OLST. In contrast, excessive fat deposition in the muscle has been associated with other physical performance measures, including weak handgrip strength [13] and knee extension strength [14], slow gait speeds [13, 15], prolonged chair standing time [15], and faster gait speed decline [16]. If OLST was associated not only with muscle mass but also with fat deposition in the muscle similar to another physical performance measure, these results support to use OLST as a simple measure of physical performance.

Given the above, we investigated an association between mid-thigh skeletal muscle mass and fat deposition in the muscle, which we determined through computational tomography image analysis, and OLST to clarify related muscle characteristics. We also investigated associations with center-of-gravity fluctuations, which we measured using a posturography, to clarify whether the associations with muscle mass and intramuscular fat deposition are specific to the method used to measure postural instability.

## Methods

### Study setting

This study was conducted in a cross-sectional setting using data from the local population.

### Study participants

Study participants were community residents who voluntarily participated in a health check-up program conducted by the Anti-aging Center of Ehime University Hospital [17, 18]. This program is provided for community residents without any requirement for participation to evaluate factors relating to cardiovascular disease, dementia, and death. Participants who gave informed consent for using their clinical information obtained at the health check-up program for a longitudinal study were enrolled in the Shimanami Health Promoting Program (the J-SHIPP study) conducted by Ehime University Graduate School of Medicine.

Of the 2244 community residents who participated in the health check-up program between February 2006 and March 2023, mid-thigh computed tomography images and postural instability measurements were obtained for 1983. We excluded participants younger than 65 (*N* = 836) or whose clinical values deviated significantly from this analysis population’s distribution [center-of-gravity path length (eyes open) ≥ 400 cm: *N* = 1, and circumferential area (eyes closed) ≥ 40 cm^2^: *N* = 2], leaving 1144 participants. The analysis used the earliest measurement for those who repeatedly participated in the health checkups.

All study procedures were approved by the Ethics Committee of Ehime University Graduate School of Medicine (30-K6). Written informed consent was obtained from all participants.

### Study procedure

The health check-up program was performed in the morning at the Ehime University Hospital. Participants were required to fast for at least 12 h prior to participation. Clinical measurements, such as anthropometric parameters, cardiovascular parameters, cognitive function, and physical performance including OLST and fluctuations of the center of gravity, as well as blood specimen sampling, were performed on the same day, while computed tomography and magnetic resonance imaging were performed on a different day with 18 ± 10 days interval. Imaging examinations were performed only on participants who requested the examination.

### Mid-thigh skeletal muscle mass and quality

Computed tomography measurements for the mid-thigh and abdomen at the check-up program were provided upon the patient’s request. Unenhanced computed tomography images were obtained at the midpoint between the lower margin of the femoral condyles and the upper margin of the greater trochanter using a 64-multi-detector row computed tomography (LightSpeed VCT, GE Healthcare, Tokyo, Japan). Muscle and fat cross-sectional areas (CSA) at the mid-thigh (sum of both legs) were measured using an image analysis software, Image J, in the following setting: normal-density fat, Hounsfield unit (HU) − 190 to − 30; very-low-density muscle, HU − 29 to − 1; low-density muscle, HU 0 to 34; normal-density muscle, HU 35 to 100; high-density muscle, HU 100 to 150 [19]. Total skeletal muscle CSA was calculated as the sum of very-low-density, low-density, normal-density, and high-density muscles’ CSAs (HU − 29 to 150). CSA was correlated strongly with body weight; therefore, CSA (cm^2^) divided by body weight (kg) was used for the analysis. The utility of computed tomography-measured muscle cross-sectional area in assessing skeletal muscle mass has been reported elsewhere [20]. We also computed the mean attenuation value (MAV), i.e., mean HU, of the skeletal muscle as an index of fat deposition in the muscle (myosteatosis). A lower MAV indicates greater fat deposition in the muscle [21].

### OLST

The participants selected the leg for the OLST, and they were tested with their eyes open and then closed; the examiners did not assist. Participants performed a pre-test before the OLST measurement, at which time they decided on which leg to use for the measurement by their own will. The interval until the participant lowered their raised leg (when the raised leg touched floor as a result of the participants no longer being able to tolerate standing on one leg) was measured twice with a maximum allowed time of 60 s (sec). The better of the two measurements was used for statistical analysis. The OLST is a clinical tool to assess postural instability in a static position [7]. In Japan, the test has been adopted to diagnose musculoskeletal ambulation disability symptom complex to help identify the symptoms of motor organ deterioration [7].

### Fluctuations of the center of gravity

Fluctuations of the center of gravity were measured using a posturography (Gravicorder G-5500; Anima Inc., Tokyo, Japan) with three vertical force transducers built into an equilateral triangular footplate, which was used as an index of static postural instability. Signals from the transducers were processed by a direct current amplifier and low-pass filters (cutoff frequency: 10 Hz) and stored in a computer after analog–digital conversion at a sampling rate of 20 Hz. The participants were instructed to maintain a static upright posture on the footplate with their feet together and to watch a circular achromatic target placed 200 cm ahead of their eyepoint. Data were acquired for 1 min after the subject’s posture stabilized. The subject rested for 1 min while seated, after which the measurement was repeated with the subject’s eyes closed to assess the effects of visual feedback on postural stability. Path length and circumferential area of the center of gravity moved were used as indices of postural stability. These parameters were considered to represent the neuromuscular response to maintain stability [22].

### Statistical analysis

Data are expressed as mean ± standard deviation. Quartiles of the mid-thigh CSA per body weight and MAV were calculated separately for men and women and then combined to avoid potential gender-based differences. We used analysis of variance to assess group differences concerning numeric variables and conducted a Chi-squared test to assess frequency differences. Multinominal logistic regression analysis was performed with participants who could stand on one leg for 60 s as the reference group to identify factors independently associated with OLST < 60 s, < 40 s, and < 20 s. Multiple linear regression analysis was performed to identify factors for path length and circumferential area of the center of gravity moved. All statistical analyses were performed using JMP Pro 17.1.0 software (SAS Institute Inc., Cary, NC, USA). *P* values less than 0.05 were considered as statistical significance.

## Results

**Fig. 1:**
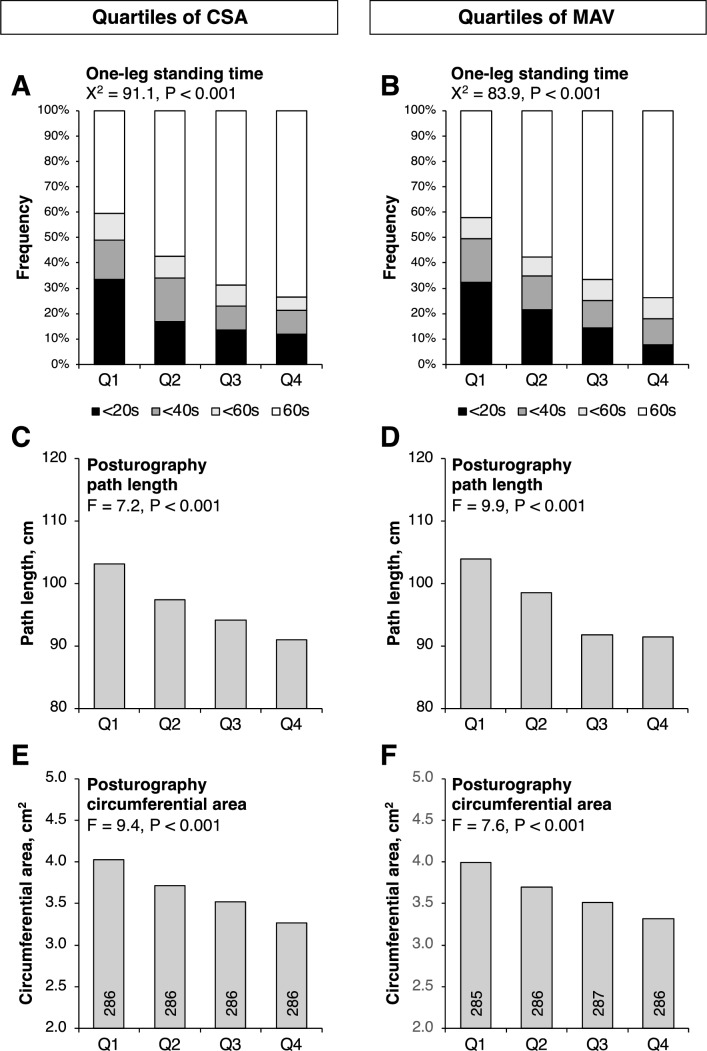
Associations between mid-thigh muscle CSA and MAV and postural instability Quartiles of mid-thigh muscle cross-sectional area (CSA) per body weight and mean attenuation value (MAV) were calculated within sex and combined to avoid potential gender differences. The lowest panel column shows the number of study participants in each quartile. Statistical significance was assessed by a Chi-squared test (**A** and **B**) or analysis of variance (**C**, **D**, **E**, and **F**)—(**A** and **B**): one-leg standing time; (**C** and **D**): posturography measured path length; (**E** and **F**): posturography measured circumference area

Table 1 shows the clinical characteristics of the study population. Figure 1 shows associations between mid-thigh muscle CSA and MAV quartiles and postural instability assessed with eyes open. A linear inverse correlation was found between muscle properties and OLST (Fig. 1A and 1B); shorter OLST indicated smaller muscle CSA and lower MAV. The CSA and MAV quartiles were also inversely associated with path length (Fig. 1C and 1D) and circumference area (Fig. 1E and 1F) of the center of gravity moved. Despite worsening instability parameters when measured with eyes closed, similar inverse associations were observed with the muscle CSA and MAV (Supplementary Fig. S1).

**Table 1:** Clinical characteristics of the study participants (*N* = 1144)

Age, years	71.8 ± 4.9
Sex, men	477
Body mass index, kg/m^2^	23.1 ± 3.0
***Mid-thigh skeletal muscle CSA***	
Normal-density fat, cm^2^	130 ± 50
Total skeletal muscle, cm^2^	217 ± 48
Very low-density muscle, cm^2^	11 ± 4
Low-density muscle, cm^2^	36 ± 13
Normal-density muscle, cm^2^	166 ± 41
High-density muscle, cm^2^	3 ± 2
***Mid-thigh skeletal muscle MAV***	
Total skeletal muscle, HU	49 ± 5
***Center-of-gravity fluctuations***	
Eyes open	
Path length, cm	96 ± 33
Circumferential area, cm^2^	3.6 ± 1.8
Eyes closed	
Path length, cm	154 ± 73
Circumferential area, cm^2^	6.2 ± 4.2
***One-leg standing time***	
Eyes open (60/ < 60/ < 40/ < 20 s), n	687/93/147/217
Eyes closed (60/ < 60/ < 40/ < 20 s), n	12/25/84/1,023

Values are mean ± standard deviation or number. Muscle and fat cross-sectional areas (CSA) were measured using computed tomography images of the mid-thigh. The mean attenuation value (MAV) of the mid-thigh skeletal muscle (very-low-, low-, normal-, and high-density muscles) was used as an index of fat deposition in the muscle. Fluctuations of the center of gravity were measured using posturography. Standing on one leg was measured with a maximum of 60 s. *HU* indicates hounsfield unit

**Fig. 2:**
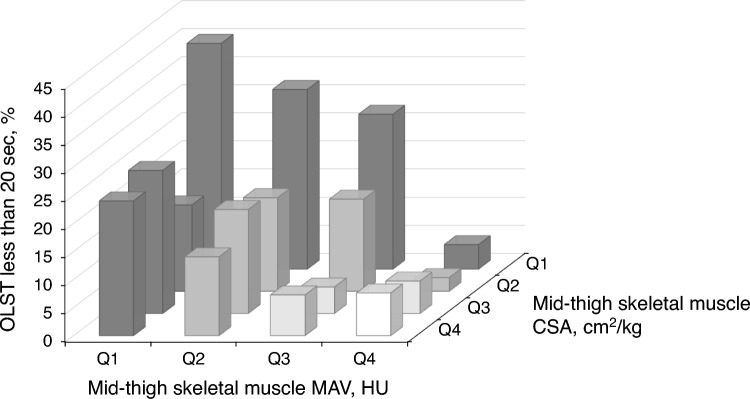
Frequency of participants with a one-leg standing time of less than 20 s Quartiles of mid-thigh muscle cross-sectional area (CSA) per body weight and mean attenuation value (MAV) were calculated within sex and combined to avoid potential sex differences. HU hounsfield unit, OLST one-leg standing time

We then performed multinominal logistic regression analysis for OLST with eyes open to clarify whether the associations of muscle CSA and MAV were independent of other covariates. Table 2 summarizes the analysis results, indicating that muscle CSA and MAV were independently associated with OLST less than 20 s when the subgroup consisting of participants who could stand more than 60 s was used as a reference. Similar results were observed when muscle CSA (< 60 s: coefficient = –0.40, *P* = 0.178, < 40 s: coefficient = –0.63, *P* = 0.012, < 20 s: coefficient = –0.78, *P* = 0.001) and MAV (< 60 s: coefficient = –0.02, *P* = 0.583, < 40 s: coefficient = –0.05, *P* = 0.038, < 20 s: coefficient = –0.09, *P* < 0.001) were included in the continuous variables. Figure 2 shows the frequency of participants who could not stand on one leg for more than 20 s. The CSA and MAV were independently associated with OLST; therefore, the frequency increased additively with the combination of CSA and MAV quartiles. Among the participants who could stand on one leg for less than 20 s (*N* = 217), 65 participants belonged to the worst subgroup of muscle properties (lowest quartiles of both CSA and MAV), while the other group (OLST ≥ 20 s, *N* = 927) inducted 97 participants. The crude odds ratio of individuals with OLST below 20 s for belonging the worst subgroup was 4.19 (95% confidence interval: 3.01 − 5.84).

**Table 2:** Multinominal logistic regression analysis for one-leg standing time with eyes open

	< 60 s		< 40 s		< 20 s	
	Coefficient	*p*	Coefficient	*p*	Coefficient	*p*
Age, years	0.136	< 0.001	0.150	< 0.001	0.229	< 0.001
Sex, men	0.064	0.782	0.204	0.292	− 0.334	0.073
Body mass index, kg/m^2^	0.115	0.007	0.055	0.122	0.059	0.070
***Skeletal muscle***						
*Cross-sectional area, cm*^*2*^*/kg*						
Q1	Reference		Reference		Reference	
Q2	− 0.486	0.117	− 0.172	0.501	− 0.911	< 0.001
Q3	− 0.499	0.126	− 0.656	0.025	− 0.874	0.001
Q4	− 0.861	0.020	− 0.568	0.060	− 0.831	0.002
*Mean attenuation value, HU*						
Q1	Reference		Reference		Reference	
Q2	− 0.198	0.552	− 0.368	0.154	− 0.389	0.090
Q3	0.065	0.846	− 0.495	0.073	− 0.632	0.012
Q4	0.183	0.600	− 0.522	0.072	− 1.203	< 0.001

Multinomial logistic regression analysis was performed with participants who could stand on one leg for 60 s as the reference group. The remaining participants were sub-grouped by one-leg standing time as < 60 s (≥ 40 and < 60 s), < 40 s (≥ 20 and < 40 s), and < 20 s. Quartiles of mid-thigh skeletal muscle cross-sectional area (per body weight) and mean attenuation value were calculated within gender and combined to avoid potential sex differences. *HU* hounsfield unit

**Fig. 3:**
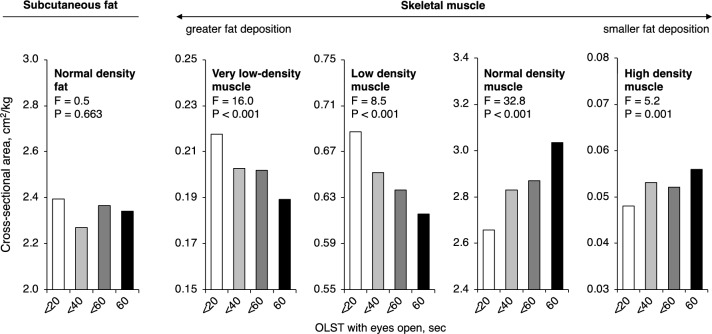
Associations between one-leg standing time and different types of mid-thigh muscle CSA Values are mean. Cross-sectional areas (CSA) per body weight of subcutaneous fat and skeletal muscles at the mid-thigh were calculated separately by the density (Hounsfield unit, HU) on computed tomography images. Normal-density fat: − 190 to − 30 HU; very-low-density muscle: − 29 to − 1 HU; low-density muscle: 0 to 34 HU; normal-density muscle: 35–100 HU; high-density muscle: 101–150 HU. The study participants were sub-grouped by one-leg standing time as 60 s, < 60 s (≥ 40 and < 60 s), < 40 s (≥ 20 and < 40 s), and < 20 s. Statistical significance was assessed by analysis of variance. OLST one-leg standing time

We evaluated the correlation between OLST and muscle CSA separately for each muscle type (Fig. 3) to determine how muscle MAV was related to OLST independently of muscle CSA. The CSA of muscles with greater fat deposition that showed lower attenuation value on computed tomography images were inversely associated with OLST. In contrast, CSA of normal and high-density muscles with smaller fat deposition showed a positive association with OLST. Because muscle MAV is an average of the attenuation values of very-low-density, low-density, normal-density, and high-density muscles, MAV may represent different muscle properties regarding OLST than CSA.

Supplementary Table S1 summarizes the multiple linear regression analysis results for the posturography parameters measured. The muscle CSA showed a weak association with the circumference area of the center-of-gravity movement measured with eyes closed; however, no clear tendency was observed.

## Discussion

This cross-sectional study in an older general population clarified that OLST was inversely associated with mid-thigh skeletal muscle mass and fat deposition in the muscle; however, no clear relationship was observed with the center-of-gravity fluctuations. The OLST is a simple and easily measurable physical performance measure that represent both loss of skeletal muscle mass and reduces muscle quality.

 Previous studies reported muscle mass decline and intramuscular fat deposition associated with physical performance measures of lower extremities, such as knee extension strength [14], gait speeds [13, 15, 16], and prolonged chair standing time [15]. Although shorting OLST has been thought to occur due to muscle weakness or impairment of sensory, motor, and central processing systems, our results suggested the importance of recognizing OLST as a manifestation of poor muscle quality. Fluctuations in the center of gravity measured in a static position did not correlate with muscle properties. Poor muscle characteristics would not be assessed by static balance measurements that do not require muscular exertion.

In the range-specific analysis of MAV, OLST was positively correlated with less-fat muscle CSAs, while inversely correlated with fatty muscle CSAs. Total muscle CSA is the sum of low-fat to fatty muscle CSAs; thus, MAV, which reflects differences in skeletal muscle components, would have shown an independent association with OLST. Most mid-thigh muscle CSA was composed of normal-density muscles. Conversely, increases in very-low-density and low-density muscles, which only share 20 percent of mid-thigh muscle CSA, can significantly affect physical performance. The lack of association between mid-thigh subcutaneous fat and OLST supports the importance of fat infiltration into the muscle and not ectopic fat accumulation in other regions (even though it shares a small portion of the skeletal muscle mass) as a factor in worsening physical performance measures.

Obesity is a primary reason for increasing ectopic fat accumulation. The same is true for intermuscular fat accumulation, as we previously reported that body mass index was a robust inverse determinant for MAV [18]. Exercise intervention has been consistently reported to reduce muscular fat accumulation [23], and favorable effects were observed across a wide range of ages and various types of exercise and physical activity interventions. Furthermore, a combination of weight loss and exercise intervention significantly reduced intermuscular adipose tissue, though diet-induced weight loss alone did not sufficiently reduce the adipose tissue [24]. We did not clarify whether interventions can reduce fat accumulation in the muscle and consequently increase OLST. However, given the results of previous studies, interventions based on exercise but not weight loss alone could have a positive effect on OLST, although further study is needed on this issue.

Guidelines from the Asian Working Group for Sarcopenia [4] state that, in the primary health care or community preventive services settings, individuals suspected of having “possible sarcopenia” measured by muscle strength (grip strength) or physical ability (five chair stand test) require aggressive intervention, including consultation with a geriatrician without measuring of muscle mass. This algorithm can help promote sarcopenia screening in the community by identifying individuals at risk for sarcopenia without using any specific device. This study found that the frequency of individuals in the lowest quartiles of both CSA and MAV was approximately three times as high among individuals who stood on one leg for less than 20 s. Although direct comparison among OLST, grip strength, and 5-time chair stand test concerning skeletal muscle mass and muscle quality is needed, the present results indicate the possibility of using OLST as an alternative measure in assessing the possibility of sarcopenia.

The strength of this study was the availability of computed tomography-assessed objective measures of mid-thigh muscle CSA and MAV, which revealed the usefulness of OLST in assessing muscle mass and muscle quality. A major limitation of this study was the lack of other physical performance measures for the entire population, making it difficult to compare the superiority of OLST to other measures.

In conclusion, short OLST was independently associated with both muscle mass loss and muscle quality degradation in older community residents similar to another physical performance measure. These results support to use OLST as a simple measure of physical performance.

### Supplementary Information

Below is the link to the electronic supplementary material.

Supplementary file1 (DOCX 171 KB)
