# Supplementary material for: One-leg standing time is a simple measure for loss of skeletal muscle mass and fat deposition in muscle: the J-SHIPP study
Source: Aging Clin Exp Res. 2024 Jan 28;36(1):7. doi: 10.1007/s40520-023-02665-8 (PMC10821963; doi:10.1007/s40520-023-02665-8)
Supplement: Supplementary file 1 — Supplementary file1 (DOCX 171 KB) [file 40520_2023_2665_MOESM1_ESM.docx]

# Supplementary materials

One-leg standing time is a simple measure for

loss of skeletal muscle mass and fat deposition in muscle

the J-SHIPP study

# Correspondence

Yasuharu Tabara

Graduate School of Public Health,

Shizuoka Graduate University of Public Health

Kita-ando 4-27-2, Aoi-ku, Shizuoka 420-0881, Japan

Tel: +81-54-295-5400, Fax: +81-54-248-3520

E-mail: tabara@s-sph.ac.jp

**Supplementary Table 1**. Multiple linear regression analysis for fluctuations in the center of gravity

|  | Eyes open | | | | |  | Eyes closed | | | | |
| --- | --- | --- | --- | --- | --- | --- | --- | --- | --- | --- | --- |
|  | Path length | |  | Circumferential area | |  | Path length | |  | Circumferential area | |
|  | β | p |  | β | p |  | β | p |  | β | p |
| Age, years | 0.321 | <0.001 |  | 0.232 | <0.001 |  | 0.234 | <0.001 |  | 0.177 | <0.001 |
| Sex, men | 0.161 | <0.001 |  | 0.157 | <0.001 |  | 0.204 | <0.001 |  | 0.222 | <0.001 |
| Body mass index, kg/m^2^ | 0.078 | 0.012 |  | 0.039 | 0.217 |  | <0.001 | 0.993 |  | −0.052 | 0.102 |
| ***Skeletal muscle*** |  |  |  |  |  |  |  |  |  |  |  |
| *Cross-sectional area, cm^2^/kg* |  |  |  |  |  |  |  |  |  |  |  |
| Q1 | reference | |  | reference | |  | reference | |  | reference | |
| Q2 | −0.036 | 0.292 |  | −0.050 | 0.151 |  | −0.024 | 0.487 |  | −0.096 | 0.006 |
| Q3 | −0.019 | 0.603 |  | −0.054 | 0.144 |  | −0.048 | 0.192 |  | −0.087 | 0.019 |
| Q4 | −0.034 | 0.366 |  | −0.093 | 0.015 |  | −0.034 | 0.380 |  | −0.100 | 0.009 |
| *Mean attenuation value, HU* |  |  |  |  |  |  |  |  |  |  |  |
| Q1 | reference | |  | reference | |  | reference | |  | reference | |
| Q2 | −0.018 | 0.598 |  | −0.027 | 0.438 |  | −0.032 | 0.362 |  | −0.034 | 0.330 |
| Q3 | −0.065 | 0.067 |  | −0.038 | 0.291 |  | −0.051 | 0.162 |  | −0.043 | 0.234 |
| Q4 | −0.040 | 0.273 |  | −0.057 | 0.136 |  | −0.053 | 0.163 |  | −0.080 | 0.036 |

Quartiles of mid-thigh skeletal muscle cross-sectional area (per body weight) and mean attenuation value were calculated within sex and then combined to avoid potential sex differences. HU: Hounsfield unit.

**Supplementary Figure 1**. Associations between mid-thigh muscle CSA and MAV and postural instability with eyes closed

Quartiles of mid-thigh muscle cross-sectional area (CSA) per body weight and mean attenuation value (MAV) were calculated within sex and then combined to avoid potential sex differences. Numbers of study participants in each quartile are shown in the column of the lowest panel. Statistical significance was assessed by a chi-squared test (A and B) or analysis of variance (C, D, E and F). **A** and **B**: one-leg standing time, **C** and **D**: posturography measured path length, **E** and **F**: posturography measured circumference area.
